# An unusual finding after adrenal surgery: a case series of adrenal schwannomas

**DOI:** 10.3389/fsurg.2023.1175633

**Published:** 2023-05-24

**Authors:** Mehmet Kostek, Mehmet Taner Unlu, Ozan Caliskan, Nurcihan Aygun, Yalin Iscan, Ahmet Cem Dural, Ismail Cem Sormaz, Fatih Tunca, Yasemin Giles Senyurek, Mehmet Uludag

**Affiliations:** ^1^ Department of General Surgery, University of Health Sciences, Sisli Hamidiye Etfal Training and Research Hospital, Istanbul, Türkiye; ^2^ Department of General Surgery, Istanbul Faculty of Medicine, Istanbul University, Istanbul, Türkiye; ^3^ Department of General Surgery, Faculty of Medicine, Istinye University, Istanbul, Türkiye

**Keywords:** adrenal glands, schwannoma, adrenalectomy, adrenocortical carcinoma, adrenal schwannoma

## Abstract

Adrenal schwannomas are rare benign tumors with no specific imaging and laboratory findings to diagnose preoperatively. Due to the limited number of cases in the literature, clinical, imaging, and pathological findings are presented in this study. Case 1 is a 61-year-old woman patient who has a 31-mm mass in the right adrenal gland. This mass was nonfunctional; in imaging studies, this mass had a cystic necrotic component, and high 18-fluorodeoxyglucose (FDG) uptake was seen. There was no metaiodobenzylguanidine (MIBG) uptake. Laparoscopic transabdominal right adrenalectomy was performed, and the pathology result was consistent with adrenal schwannomas. Case 2 is a 63-year-old man patient who presented with a 38-mm mass in the left adrenal gland. This mass was nonfunctional and similar to that in Case 1; this mass had a cystic component. Laparoscopic transabdominal left adrenalectomy was performed. The diagnosis of adrenal schwannoma with degeneration was revealed. Case 3 was a 72-year-old woman patient admitted to the hospital for a 125-mm left adrenal mass. Similar to Case 1, this mass also had a cystic necrotic component in imaging studies. High FDG uptake was seen, and the patient underwent conventional adrenalectomy due to the suspicion of malignancy. After pathological evaluation, a diagnosis of adrenal schwannoma was made. A main diagnostic challenge in adrenal schwannomas is the preoperative diagnosis. These masses have no pathognomonic finding or specific hormonal function. Imaging findings of these masses may increase the suspicion of malignancy, which may affect decisions for surgery and the surgical technique.

## 1. Introduction

Schwannomas are mostly benign tumors that comprise Schwann cells neighboring to myelinated parts of central or peripheral neurons. These rare tumors generally appear at flexor surfaces of extremities, mediastinum, head, and neck regions. Infrequently, these tumors are seen in the retroperitoneal area (1, 2). Retroperitoneal schwannomas constitute 1% of all retroperitoneal masses (3).

Common benign tumors originating from adrenal tissue are benign adenomas and pheochromocytomas; however, schwannomas rarely originate from adrenal tissue and are considered as only 0.7% of benign adrenal tumors (4). Due to the lack of pathognomonic findings, preoperative diagnosis is challenging, and postoperative pathology results are needed for the final diagnosis.

Adrenal schwannomas may be confused with adrenocortical cancer, adrenal metastasis, or pheochromocytoma due to their imaging findings (5). In this case series, we present characteristics, preoperative clinical, radiologic, and postoperative findings of three different adrenal schwannomas with suspicious findings in imaging studies for malignancy or pheochromocytoma; however, after adrenal surgery, the diagnosis of benign adrenal schwannoma was revealed in pathological evaluation.

## 2. Case reports

### 2.1. Case 1

**Figure 1:**
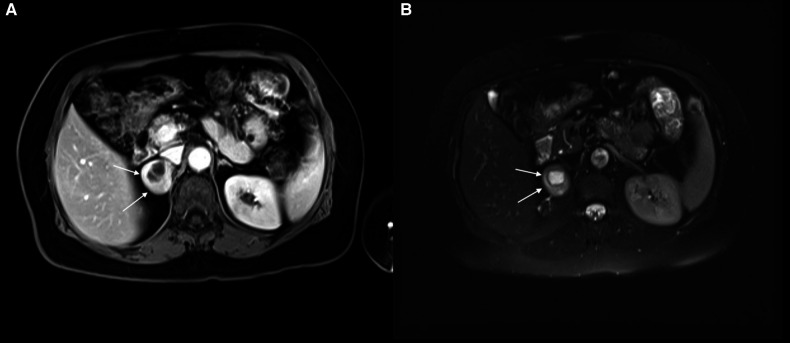
(**A**) right adrenal schwannoma in the T1 sequence of MRI. The cystic necrotic component can be seen in this imaging study. The schwannoma was shown by a white arrow. (**B**) Right adrenal schwannoma in the T2 sequence of MRI. The schwannoma was shown by a white arrow.

**Figure 2:**
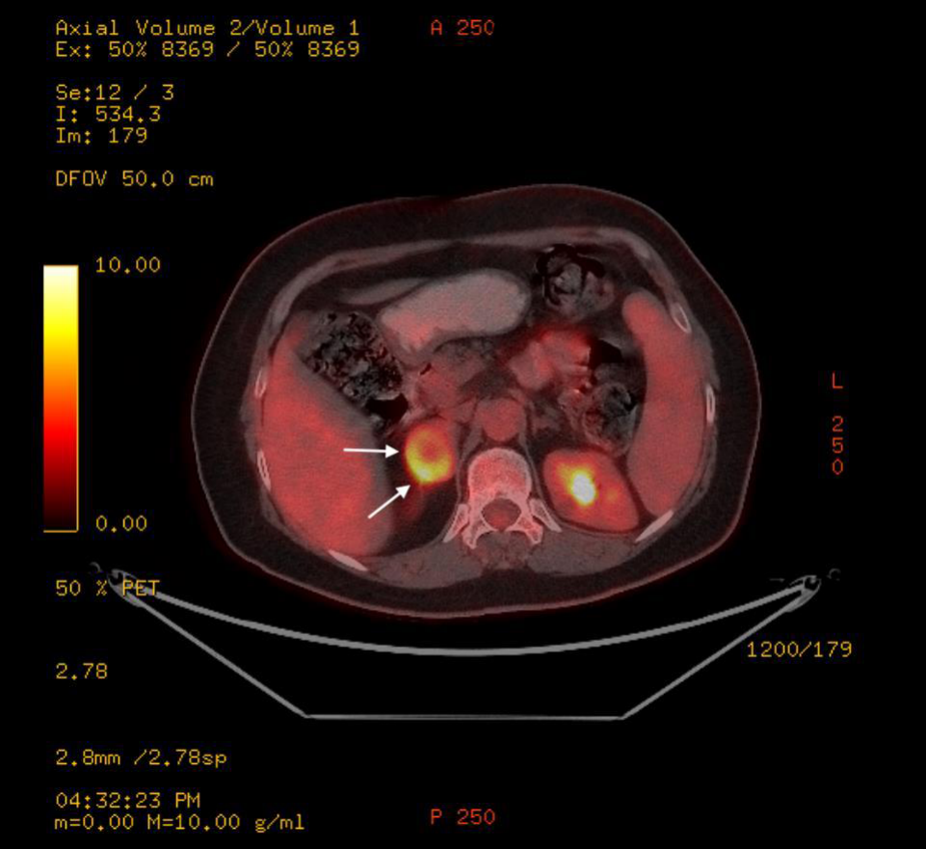
Right adrenal schwannoma in the FDG-PET/CT scan. This mass had an increased FDG uptake. The schwannoma was shown by a white arrow.

A 61-year-old woman patient was admitted to the outpatient clinic because of a 31-mm mass in the right adrenal gland after undergoing a computed tomography (CT) scan for COVID-19. The patient has no medical history other than COVID-19. The CT scan showed a cystic necrotic component that caused suspicion of adrenocortical carcinoma or pheochromocytoma. The patient was evaluated for excess hormone production with the 1-mg dexamethasone suppression test, serum and urine metanephrines, plasma renin activity, and serum aldosterone. There was no excess hormone production by the adrenal mass. Several imaging studies, in addition to computed tomography scans, were performed. Magnetic resonance imaging (MRI) showed a semisolid mass with cystic and necrotic components and peripheral diffusion restriction, which increased suspicion of adrenocortical carcinoma (Figures 1A,B). Therefore, the patient underwent 18-fluorodeoxyglucose positron emission tomography combined with computed tomography (FDG-PET/CT). In this imaging study, an enhancement of FDG at the level of malignancy was seen, and the maximum standardized uptake value (SUVmax) was 9.94 (Figure 2). Due to heterogeneous imaging findings, to exclude silent pheochromocytoma or any metastasis of pheochromocytoma, the patient also underwent metaiodobenzylguanidine (MIBG) scintigraphy; however, there was no enhancement throughout body.

**Figure 3:**
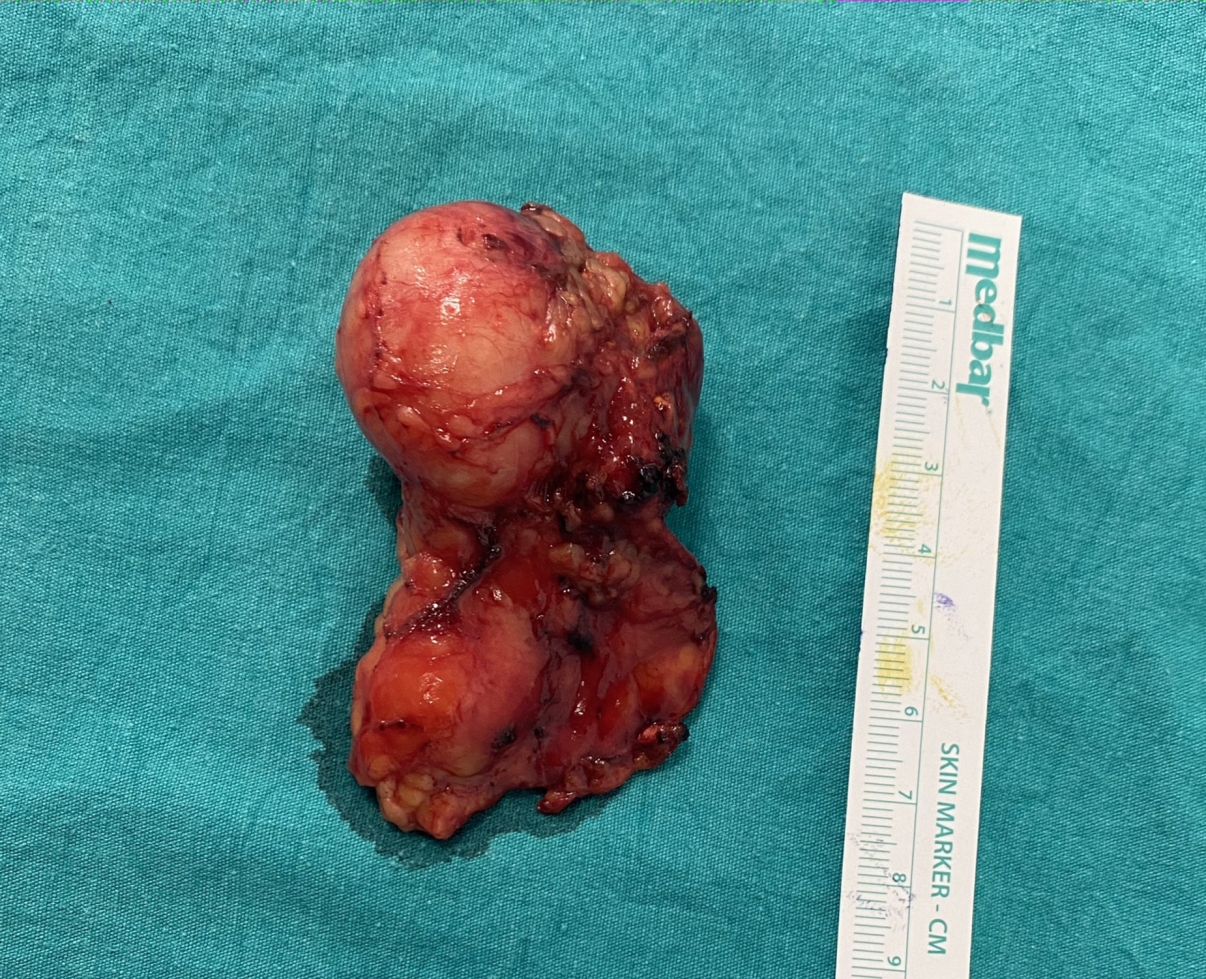
Right adrenal schwannoma after laparoscopic transabdominal right adrenalectomy. Two components of adrenal mass can be observed.

Owing to the suspicious findings in the imaging studies, surgical excision was planned. Because of the small mass size, laparoscopic transabdominal adrenalectomy was applied. During gross examination of the excised material, two components were easily seen (Figure 3). One cystic component with suspected mass and unaffected adrenal gland. After the operation, the patient had nothing remarkable and was discharged postoperative on the third day. Postoperative follow-up was continued until 8 months, and there was no recurrence or any complaints after surgery.

At the end of the pathological examination, the final diagnosis was consistent with adrenal schwannoma. Needle-shaped cells without atypia were seen under a microscope, and diffuse positive reactivity with S100 protein was seen in immunohistochemistry. There was no staining with CD34, Desmin, and SMA. The Ki-67 index was less than 1%.

### 2.2. Case 2

A 63-year-old man patient was admitted to the general surgery clinic because of a left upper quadrant pain. The patient had no history of medical illnesses and no history of abdominal surgery. After an unremarkable routine physical examination, abdominal ultrasound imaging was ordered. Ultrasound imaging revealed a 38-mm × 36-mm mass in the left adrenal gland with a cystic component surrounded by a thick wall. A biochemical investigation was planned, and the patient was evaluated for excess hormone production with the 1-mg dexamethasone suppression test, serum and urine metanephrines, plasma renin activity, and serum aldosterone. There was no hormonal excess, and no hormone production by the adrenal mass was shown. The patient underwent an abdominal MRI scan, and a thick-walled adrenal mass with a cystic component was revealed. The size of the mass was 33 mm × 37 mm, and the mass was described as isohyperintense in T1-weighted images, hypo-hyperintense in T2-weighted images, and contrast enhancement at the postcontrast series. Due to its heterogeneous imaging characteristics, laparoscopic left transabdominal adrenalectomy was performed. The patient had an unremarkable hospital stay and was discharged on the third day postoperatively. The final diagnosis of the adrenal mass was concluded as schwannomas with degeneration in the pathology report. The patient continued to follow-up examinations for 12 years and had nothing remarkable in imaging studies and biochemical evaluations.

### 2.3. Case 3

**Figure 4:**
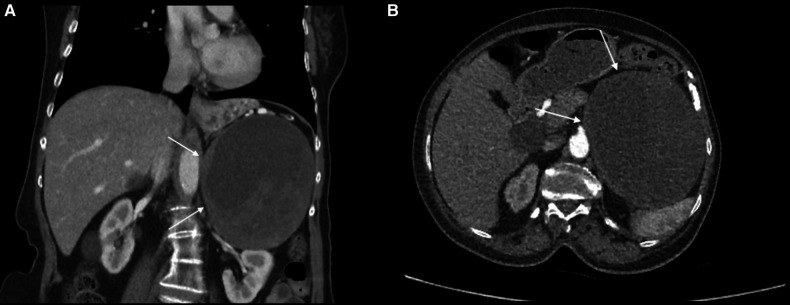
(**A**) left adrenal schwannoma in the coronal plane of the CT scan. The cystic necrotic component can be seen in this imaging study. The schwannoma was shown by a white arrow. (**B**) Left adrenal schwannoma in the axial plane of the CT scan. The cystic necrotic component can be seen in this imaging study. The schwannoma was shown by a white arrow.

A 72-year-old woman patient was presented to the outpatient clinic for left adrenal incidentaloma. This mass appeared in a CT scan during the investigation for hydrocephalus. The patient has a past medical history of high blood pressure, and vertigo, and laparoscopic cholecystectomy. In routine laboratory tests, no excess hormone production was shown. In the CT scan, the size of the left adrenal mass was 125 mm × 102 mm × 123 mm, and heterogeneous contrast enhancement with cystic-necrotic components was observed (Figures 4A,B). This mass was pushing the pancreas and splenic vein to the anterior, and it was in close contact with the stomach; however, there was no sign of invasion. In whole-body FDG-PET/CT, FDG uptake was observed on the adrenal mass, and SUVmax was 7.1. This finding increased suspicion of malignancy. Because of its size and suspicion of malignancy, conventional adrenalectomy was applied. The operation was uneventful, and the patient was discharged on the 8th day postoperatively without any complications. The final pathology result was consistent with schwannomas, and staining with immunohistochemistry was positive for S100 and negative for SMA, CD34, Desmin, and CD117. The Ki-67 score was 1%−2%. Ten months after the operation, the patient has no complaint in the follow-up examinations, and no recurrence has been observed.

## 3. Discussion

Schwannomas are frequently seen in the head, neck, and extremities; however, adrenal schwannomas are a rare clinical entity and constitute only 0.7% of benign adrenal tumors (4). Schwannomas were mostly seen as sporadic cases (almost 90%); on the other hand, several cases associated with neurofibromatosis type 2, Carney complex, and schwannomatosis were reported (1, 6). Nevertheless, none of the adrenal schwannomas described to date have been associated with genetic syndromes (7).

Adrenal schwannoma cases are seen more frequently at ages 39 and 59 years and more frequently in women (1.17 times more than men) (1, 6). These masses were mostly seen in the 5th and 6th decades and more often in women. However, two of our cases were in their 7th decade and one of them was in her 8th decade. In a literature review by Incampo and colleagues, 169 patients with adrenal schwannomas were reviewed, and approximately 25% of patients were in their 7th decade or older (7).

The largest diameters of the previously reported adrenal schwannomas varied between 1 and 15 cm, and 64% of these masses were between 4 and 10 cm (7). However, in our study, the largest diameters of Cases 1 and 2 were less than 4 cm and the largest diameter of Case 3 was 12.5 cm. In nonhormone-secreting masses of the adrenal gland, the possibility of malignancy is the main indication for surgery, and suspicious imaging findings guided the surgeons for adrenalectomy in Cases 1 and 2. In Case 3, the largest diameter was more than 6 cm, and consequently, higher suspicion for malignancy was raised due to the increased size. After the operation, none of the masses were concluded as malignant tumors.

Patients with adrenal schwannomas mostly have complaints of vague pain on the left upper quadrant or have no complaint and incidentally detected a mass on adrenal glands. Due to the COVID-19 pandemic, many patients underwent thorax CT scans, which may increase the possibility of finding more incidentalomas. However, adrenal schwannomas cannot be diagnosed from imaging studies and biochemical tests. Hormonal inactivity of these masses may not be helpful to clinicians for their differential diagnosis; nevertheless, imaging findings may confuse clinicians for preoperative diagnosis. Heterogeneous imaging findings such as thick walls, mixed cystic solid components, calcification, and necrosis may cause suspicion of pheochromocytoma and adrenocortical carcinoma (5). In MRI studies, intensity characteristics of adrenal schwannomas may be low in T1-weighted images and high in T2-weighted images. However, these lesions may have heterogeneous findings as in CT scans (8) (Table 1).

**Table 1:** Radiological findings of adrenal lesions.

Radiological findings	Ultrasonography	Computed tomography	Magnetic resonance imaging	Positron emission tomography
Adrenal adenoma	Mostly less than 3 cm, hypoechoic, smooth margin, homogeneous	Homogeneous mass generally less than 4 cm, smooth margin, less than 10 HU, unilateral, rapid contrast washout	Isointense compared to liver both in T1 and T2, obvious lipid shift.	Normal metabolic activity, SUVmax of less than 5
Adrenocortical cancer	Generally, more than 6 cm, irregular margin, heterogeneous echogenicity	Mostly unilateral, larger than 4 cm, irregular margin, central necrosis, slow contrast washout	Hypointense compared to liver on T1, medium-to-high signal intensity on T2	Increased metabolic activity, mostly SUVmax of more than 5
Pheochromocytoma	Generally, more than 5 cm, increased vascularity, hypoechoic or mixed echogenicity	Cystic and/or hemorrhagic changes, increased vascularity, more than 20 HU, slow contrast washout	Increased signal intensity on T2	Increased metabolic activity, mostly SUVmax of more than 5
Adrenal metastases	Irregular margin, heterogeneous echogenicity	Heterogenous with irregular margin, unilateral or bilateral, more than 20 HU, slow contrast washout	Isointense compared to liver on T1, medium-to-high signal intensity on T2	Increased metabolic activity, mostly SUVmax of more than 5
Schwannoma	Solid, hypoechoic lesions with smooth margins, the internal structure may be heterogeneous with cystic degeneration, and the hyperechoic thin capsule may be present	Smooth, solid lesions, mostly more than 15 HU, heterogeneous with cystic components. mild contrast enhancement, slow washout	Low signal intensity on T1 and heterogeneous high signal intensity on T2	Heterogeneous increased metabolic activity

HU, Hounsfield unit; SUVmax, maximum standardized uptake value (4, 7, 9, 10).

In addition to the other imaging techniques, FDG-PET/CT scans may give information about the metabolic activity of adrenal lesions (11). Adrenal schwannomas may show increased FDG uptake, and evaluation of this imaging study may increase suspicion of malignancy. In previous reports, only seven cases of adrenal schwannoma were reported with the results of the PET scan, and they were presented with increased FDG uptake (7). In this study, two of our patients were presented with increased FDG uptake. In Case 1, the adrenal mass had increased FDG uptake and cystic necrotic component, which caused increased suspicion and led the endocrinologist and surgeons to decide on an operation. Also, the size and high FDG uptake of the mass increased suspicion of malignancy in Case 3. Other than a previously reported case of a microcystic reticular adrenal schwannoma in which the SUVmax was 71.7, the SUVmax of reported adrenal schwannomas was between 2.8 and 10.3 (1, 7, 12–14). Similar to these numbers, also in our cases, SUVmax values in Cases 1 and 3 were 9.94 and 7.1, respectively. These numbers showed high metabolic activity in these masses. The possible reasons for increased uptake are hypercellular areas in the adrenal schwannomas and peritumoral lymphoid cuffs (11). With cystic and necrotic components in the adrenal masses, positive FDG uptake may increase the suspicion of malignancy. However, at the end of the pathological evaluation, a benign result such as an adrenal schwannoma may be seen. These results show that an FDG-PET scan cannot be helpful for the differential diagnosis of schwannomas. As another whole-body screening test, MIBG scintigraphy results were rarely discussed in adrenal schwannomas. Only two cases of adrenal schwannomas previously underwent MIBG scintigraphy in the literature, and these cases had no increased uptake. Compared to the previous studies, Case 1 had an MIBG scintigraphy and had no increased uptake. Therefore, in patients with suspicious imaging findings for pheochromocytoma and without positive laboratory findings, MIBG scintigraphy can be a possible whole-body screening test to exclude pheochromocytoma (15, 16).

The main treatment modality of schwannomas is surgical excision; however, there is no certain adrenalectomy technique for schwannomas. An impression of malignancy or invasion may direct surgeons to an open adrenalectomy; therefore, excellent preoperative imaging is crucial for choosing the right surgical technique. Masses with smooth margins are suitable for laparoscopic adrenalectomy. An appropriate size of the masses for laparoscopic surgery is debatable; however, in case reports from several centers, patients with masses larger than 10 cm were operated without complications (2, 12). Pathological evaluation reveals the final diagnosis (6). Schwannomas are not sensitive to radiotherapy and chemotherapy; hence, complete excision of the mass is critical (17). Therefore, conventional adrenalectomy was chosen for Case 3. Increased possibility for malignancy in adrenal masses larger than 6 cm and increased FDG uptake played a role in this decision.

Schwannomas originate from nerve sheaths and have hypocellular and hypercellular areas of spindle cells. Immunohistochemistry results show strongly positive staining of spindle cells with S100, and this result verifies their neural origin (18). These masses are not expected to be stained by HMB45, EMA, Desmin, and SMA (2, 8). Staining with CD34 was reported in one-third of the patients (18). The Ki-67 proliferation index was reported to be less than 5% in the literature (2, 19). Due to the hypocellular and hypercellular areas of spindle cells, contrast enhancement patterns show heterogeneity, and radiological findings may be suspicious for malignancy (7). In the pathology results of these lesions, findings were concordant with the radiology reports, such as cystic changes and necrosis. However, radiological findings were suspicious of malignancy, and pathology results were consistent with increased cellularity. In all three cases, cystic changes were observed in radiological and pathological data. Although cystic and/or necrotic changes are reported in 43% of cases in the literature, focal necrotic areas were also reported in pathology results in Cases 1 and 3 (7). These results are coherent with the radiology reports.

Recurrence or metastasis is not expected after complete resection in adrenal schwannomas. There is no consensus on the follow-up period in these patients; however, long-term follow-up data are limited (2, 7, 19). There is no recurrence and distant metastasis in the cases presented in this paper, and follow-up periods are 8 months for Case 1, 12 years for Case 2, and 10 months for Case 3.

## 4. Conclusion

Adrenal schwannomas are rare tumors that are mostly diagnosed after surgical resection. Imaging findings are confounding, and these masses are nonfunctional tumors. Radiologic findings cause suspicion of malignancy, and there are no specific preoperative diagnostic criteria. The final diagnosis can be made in pathological evaluation after resection of the mass. The main treatment is surgery, and after complete resection, recurrence and metastasis are infrequent. There is limited data in the literature, and more case reports and case series are needed for better patient care.

## Data Availability

The original contributions presented in the study are included in the article; further inquiries can be directed to the corresponding author.
